# Perifosine as a Potential Novel Anti-Cancer Agent Inhibits EGFR/MET-AKT Axis in Malignant Pleural Mesothelioma

**DOI:** 10.1371/journal.pone.0036856

**Published:** 2012-05-10

**Authors:** Giulia Pinton, Arcangela Gabriella Manente, Giovanni Angeli, Luciano Mutti, Laura Moro

**Affiliations:** 1 Department of Pharmaceutical Sciences and Drug and Food Biotechnology Center, University of Piemonte Orientale A. Avogadro, Novara, Italy; 2 Service of Pathology, Vercelli Hospital, Vercelli, Italy; 3 Department of Medicine, Vercelli Hospital, Vercelli, Italy; Wayne State University School of Medicine, United States of America

## Abstract

**Background:**

PI3K/AKT signalling pathway is aberrantly active and plays a critical role for cell cycle progression of human malignant pleural mesothelioma (MMe) cells.

AKT is one of the important cellular targets of perifosine, a novel bio-available alkylphospholipid that has displayed significant anti-proliferative activity in vitro and in vivo in several human tumour model systems and is currently being tested in clinical trials.

**Methods:**

We tested Perifosine activity on human mesothelial cells and different mesothelioma cell lines, in order to provide evidence of its efficacy as single agent and combined therapy.

**Results:**

We demonstrate here that perifosine, currently being evaluated as an anti-cancer agent in phase 1 and 2 clinical trials, caused a dose-dependent reduction of AKT activation, at concentrations causing MMe cell growth arrest. In this study we firstly describe that MMe cells express aside from AKT1 also AKT3 and that either the myristoylated, constitutively active, forms of the two proteins, abrogated perifosine-mediated cell growth inhibition. Moreover, we describe here a novel mechanism of perifosine that interferes, upstream of AKT, affecting EGFR and MET phosphorylation. Finally, we demonstrate a significant increase in cell toxicity when MMe cells were treated with perifosine in combination with cisplatin.

**Conclusions:**

This study provides a novel mechanism of action of perifosine, directly inhibiting EGFR/MET-AKT1/3 axis, providing a rationale for a novel translational approach to the treatment of MMe.

## Introduction

Malignant Pleural Mesothelioma (MMe) is a rapidly lethal cancer associated with exposure to asbestos that is increasing in incidence worldwide [1], [2]. Since MMe is resistant to conventional therapies, the prognosis of these patients is poor, with a median survival of 11–12 months after diagnosis [3], [4] therefore, there is an urgent need for effective therapy.

Activation of multiple receptor tyrosine kinases (RTKs) is critical for cell proliferation and/or survival of MMe cells. Among RTKs, MET and EGFR were thought to be two of the most significantly involved in MMe proliferation and/or survival via PI3-K/AKT signalling cascade activation. However, a phase II clinical study of erlotinib treatment did not show an effect on MMe, although 96% of the specimens showed positive pEGFR [5].

The lack of EGFR mutation in MMe may be one of the reasons for the unresponsiveness [6]. MET is another RTK which mediates the activation of several signalling pathways, including phosphoinositide 3-kinase (PI3-K)/AKT and Ras/mitogen-activated protein kinase cascades [7]. Previous studies demonstrated that MET was expressed and activated in the majority of MMe cell lines and clinical specimens [8], [9]. However, MET inhibition caused growth arrest in only a small subset of MMe cell lines, regardless of frequent MET activation [10]. In a recently published paper Kawaguchi et al. suggested that inhibition of multiple RTKs may serve to develop a more effective target therapy for patients with MMe [11].

As in other cancers among the RTKs activated signals, the phosphoinositide 3-kinase (PI3K)/AKT pathway, plays a critical role for the cell cycle progression in human MMe cells [12], [13]. It has been reported that inhibition of the PI3K activity led to significant cell cycle arrest and suppression of cell proliferation of different MMe cell lines [14]. PI3K activation results in accumulation of phosphatidylinositol 3,4,5-trisphosphate and phosphatidylinositol 3,4-bisphosphate [15]. Then pleckstrin homology (PH) domain-containing proteins including PDK1 and AKT [16], [17] bind to the 3′-OH phosphorylated phosphatidylinositols through this domain. This binding results in targeting of AKT to the plasma membrane and provides a favourable conformation for AKT Thr308 and Ser473 phosphorylation [18].

The first-generation of PI3K inhibitors include LY294002 and wortmannin, both targeting the catalytic site of p110, which have been used as research tools to elucidate the value of PI3K as therapeutic target [19]. For the un-favourable pharmaceutical properties, toxicity, and cross-over inhibition of other lipid and protein kinases, they were not extensively used in clinical trials [20].

Perifosine [octadecyl-(1,1-dimethyl-piperidinio-4-yl)-phosphate] is a synthetic novel alkylphospholipid (ALP), a new class of antitumor agents which targets cell membranes of active proliferating cells and inhibits PH domain mediated AKT membrane recruitment and activation. Importantly, perifosine does not directly affect either activity of PI3K or phosphoinositide-dependent kinase 1 (PDK1) [21]. Perifosine has displayed significant anti-proliferative activity *in vitro* and *in vivo* in several human tumour model systems and is currently being tested in different clinical trials [22], [23].

The current study investigates a potential antitumor activity of perifosine using *in vitro* MMe cell models, using perifosine either on its own or in combination with established chemotherapeutic drugs. We demonstrate that perifosine inhibiting both MET and EGFR activation even in presence of HGF and EGF decreases AKT phosphorylation and blocks cell proliferation without inducing apoptosis of MMe cell lines. In this study we firstly describe that MMe cells express aside from AKT1 also AKT3 and that the perifosine induced cell growth inhibition were restored by transfection of both myristoylated-AKTs, constitutively localized to the plasma membrane. Moreover, co-treatment with perifosine substantially increases cytotoxic effect of cisplatin in MMe cells.

## Results

### Perifosine targets AKT phosphorylation and affects MMe cell proliferation inducing a G2/M phases arrest

**Figure 1:**
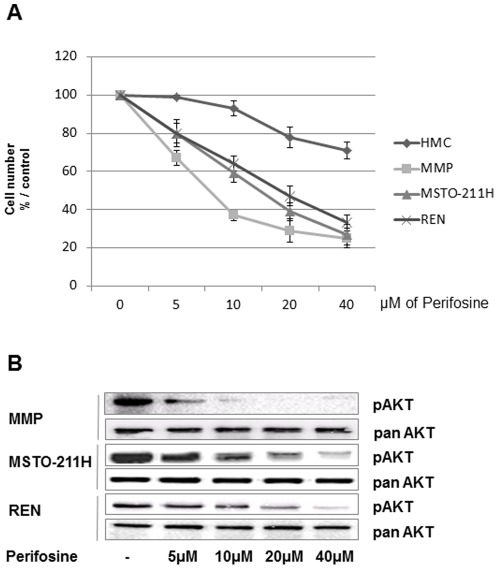
Effects of perifosine on cell viability and AKT phosphorylation. A, effect of perifosine on the viability of HMC and three different MMe cell lines (REN, MSTO211-H and MMP) after 24 hours treatment at the indicated concentrations. *Points*, means ± SD of three individual measurements. **B**, REN, MSTO211-H and MMP cells were treated with the indicated doses of perifosine for 1 hour. Cells were lysed, and equal amounts of protein were separated by SDS-PAGE, transferred to nitrocellulose membrane, probed with phospho-specific AKT and re-probed with panAKT antibodies as described in “Materials and Methods.” Representative of three independent experiments.

**Figure 2:**
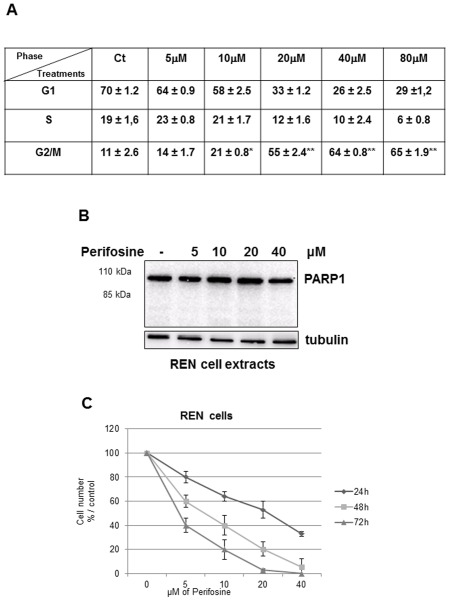
Effect of perifosine on REN cell cycle progression. A, REN cells were treated with different doses of perifosine for 24 hours. After treatments, cells were stained with propidium iodide as described in “Materials and Methods” and analysed for cellular DNA content by flow cytometry. Data reported in the table represent mean ± SD (n = 3) of the percentage of cells in each phase of the cell cycle, * p≤0.005, **p≤0.001. **B**, REN, cells were treated with the indicated doses of perifosine for 24 hours. Cells were lysed, and equal amounts of protein were separated by SDS-PAGE, transferred to nitrocellulose membrane, probed with a specific anti-PARP1 antibody and tubulin for equal loading. Representative of three independent experiments. **C**, Effect of perifosine on the viability of REN cells was tested at 24, 48 and 72 hours treatment at the indicated concentrations. *Points*, means ± SD of three individual measurements.

Perifosine has a similar structure to naturally occurring phospholipids that has been described to primarily interfere with membranes of proliferating cells like tumour cells, Here we demonstrate that 50% of Perifosine-induced MMe growth inhibition (IC50) in 24 hours was 23 µM, 14 µM and 7.5 µM for REN, MSTO211H, and MMP respectively, while minimal toxicity was displayed in HMC normal mesothelial cells (Figure 1A). These doses were in line with achieved plasma concentrations *in vivo* (described to be around 16 µM). The AKT pathway is a target for the anti-proliferative effect of perifosine, so we investigated the effect of this drug on the phosphorylation status of AKT. Figure 1B shows that exposure of MMe cells to perifosine for 1 hour caused a dose dependent loss of Ser473 phosphorylation of AKT, without affecting the total amount of the protein. This loss of AKT phosphorylation was seen in all cell lines tested at their IC50 concentration of perifosine. We elected REN cells, representative of the more common epithelioid tumours, to further characterize early signalling events affected by perifosine. In REN cells we demonstrated that the treatment with different doses of perifosine for 24 hours caused a cell cycle arrest with a progressive significant accumulation in the G2/M phases (Figure 2A). Both sub-G1 pick and cleavage of poly(ADP-ribose) polymerase were not evident upon incubation with increasing doses of perifosine for 24 h, indicating that no caspase dependent apoptotic cell death occurred (Figure 2B). Moreover, the effects of perifosine increased with time treatment leading all cells exposed to 23 µM to die by 72 hours (Figure 2C). Similar results were obtained on MSTO211-H cells (Figure S1).

### A constitutive active form of AKT over-comes perifosine inhibition

**Figure 3:**
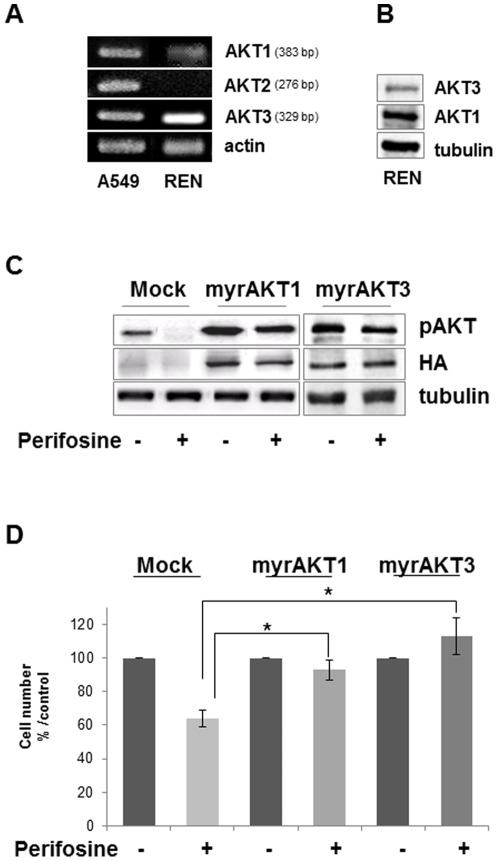
Expression of AKTs and rescue of perifosine effects by transfection with constitutive active Myr-AKTs. A, AKT1, 2, 3 and actin as control mRNA expression in A549 and REN cells was evaluated by RT-PCR as reported in in “Material and Methods” section. **B**, AKT1 and 3 protein expression was evaluated by Western blot analysis in REN cells with isoform specific antibodies, tubulin blot confirmed equal loading. Representative of three independent experiments. **C**, REN cells were transfected with 1 µg/plate of plasmid coding for the constitutive activated form of AKT, Myr-AKT1 or 3. 24 hours post transfection REN cells were treated with 23 µM perifosine for 1 hour, then were lysed and equal amounts of proteins were subjected to SDS-PAGE followed by immuno-blotting with specific antibodies to phosphorylated AKT, HA to confirm transfection and tubulin for equal loading. **D**, The effect of perifosine on cells transfected with Myr- AKT1 or 3 was also assessed on cell proliferation. REN cells were transfected with 1 µg/plate of plasmid coding Myr-AKT1 or 3 and 24 hours post transfection were treated with 23 µM perifosine for 24 hours. At the end of treatment viable cell count was determined. *Points*, means ± SD of three individual measurements. Perifosine markedly alters AKT membrane translocation, so we next asked whether forced expression of an exogenous Myr-AKT1 or Myr-AKT3 could abrogate the effect of perifosine on AKT activating phosphorylations and cell growth. Contradictory data are available in literature on the capability of a constitutively active AKT to overcome perifosine action. While in prostate cancer cells inhibition of AKT phosphorylation was substantially relieved by introduction of a Myr-AKT1, which bypassed the requirement for PH domain-mediated membrane recruitment, in myeloma cells it has been described that perifosine overcome constitutive highly active AKT1 [25], [26]. We transiently transfected REN cells either with an HA-tagged Myr-AKT1 or Myr-AKT3 and treated them for 24 hours with the IC50 dose of perifosine. Western-blot analysis reported in Figure 3C shows that transfection of both Myr-AKT1 and Myr-AKT3 overcomes perifosine inhibition of AKT phosphorylation in REN cells; only a slight reduction of phosphorylation, probably due to the block of the endogenous proteins, was observed. Cell growth analysis reported in Figure 3D demonstrates that both transfected Myr-AKTs rescued perifosine induced block of cell proliferation.

AKT comprises three closely related isoforms: AKT 1, AKT 2 and AKT 3, encoded by three different genes [24]. As the phospho-antibody doesn't discriminate between the three isoforms, in this study, AKT 1, AKT 2 and AKT 3 expression was investigated in REN cells. Data reported in Figure 3A and 3B show that REN cells express AKT1 and AKT3, while AKT2 mRNA was not evidenced (similar results on MSTO211-H cells are shown in Figure S1).

### Perifosine affects EGFR and MET signalling

**Figure 4:**
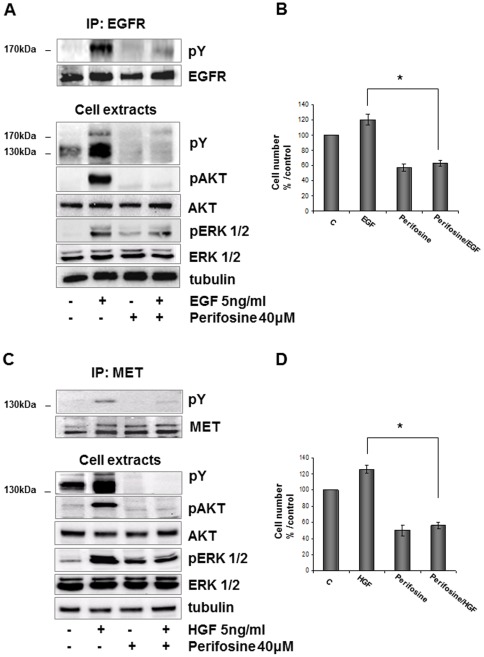
Effects of perifosine on EGFR and MET phosphorylation and signalling. A, B, Exponentially growing REN cells were pre-treated with 23 µM perifosine for 1 hour and then incubated 10 minutes with EGF (5 ng/ml) or HGF (5 ng/ml). At the end of experiment, cell lysates were prepared or immunoprecipitated and analyzed by immunoblotting with indicated antibodies. Results are representative of three different experiments. B, D, The effect of perifosine was also assessed on growth factor mediated cell proliferation. REN cells were treated with 23 µM perifosine 24 hours in the absence or in the presence of 5 ng/ml EGF or HGF. At the end of treatment viable cell count was determined. *Points*, means ± SD of three individual measurements.

Perifosine is similar to phospholipids, the main constituents of cellular membranes and can modify membrane-related signal transduction. Consistently in prostate cancer cells perifosine was able to determine a reduction of AKT activity, cell proliferation, and to induce apoptosis after stimulation with 50 ng/ml EGF, although no data concerning EGFR activation were shown so far [27]. Hence we analysed if perifosine could interfere with the ligand mediated activation of EGFR and MET by immunoprecipitation and Western-blot experiments. As shown in Figure 4A and 4C in REN cells starved, treated 1 hour with perifosine (23 µM) and then stimulated 10 minutes with 5 ng/ml of EGF or HGF both EGFR and MET were not phosphorylated even in the presence of their ligands. Also EGFR and MET signalling were significantly compromised; in fact also growth factor induced AKT phosphorylation was strongly inhibited. As described in other cell models [26], analogous concentrations of perifosine actually caused a slight increase in basal ERK1/2 phosphorylation, while counteracted that induced by growth factors. The effect of perifosine was also assessed on growth factor mediated cell proliferation. As shown in Figure 4B and 4D, 23 µM perifosine administered 24 hours to REN cells in the presence of EGF or HGF abrogated growth factor induced cell proliferation. Of note similar data were observed in the MSTO-211H cell line (Figure S1). Moreover, perifosine was more effective than single or combined usage of EGFR and MET specific inhibitors, either in terms of cell viability than of AKT activation (data not shown).

### Perifosine enhances response of MMe cells to chemotherapeutic agents

**Figure 5:**
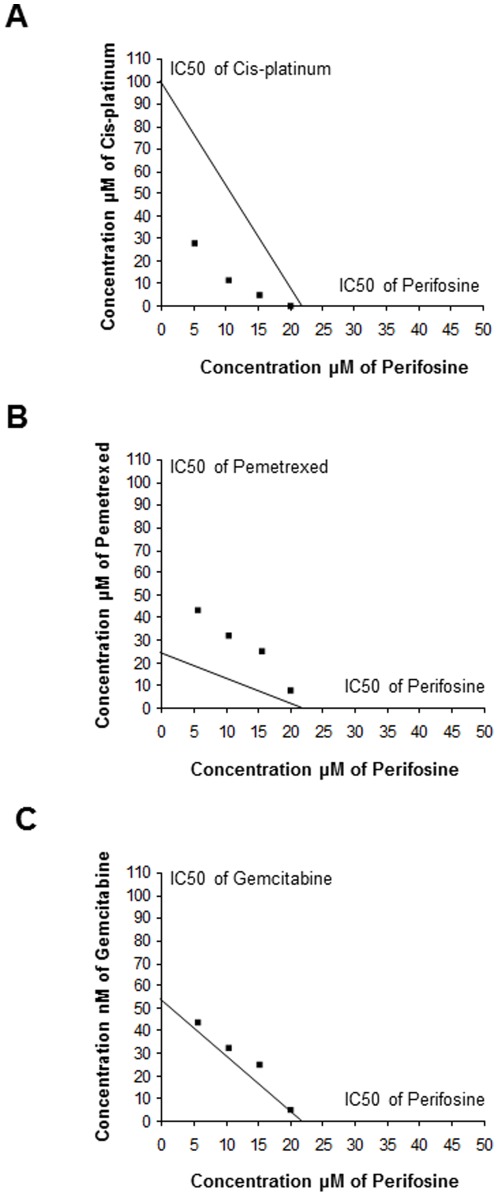
Isobologram analysis of drug interactions. A, B, C, Isobologram plots of the interactions between perifosine and cisplatin or gemcitabine or pemetrexed compounds on REN cells. Cells (5×10^4^) were plated in complete medium and allowed to adhere to the surface overnight. The plating medium was removed and replaced with medium containing various concentrations of perifosine and the IC50 doses of the other drugs. To determine the nature of the interaction between perifosine and cis-platinum or gemcitabine or pemetrexed, we counted surviving cells following 24 hours drug incubation. The diagonal line represents the isoeffect line of additivity. Each point is the mean determined from experiments performed in triplicate.

To determine the nature of interactions between perifosine and chemotherapeutic agents currently used in the MMe therapy, we examined data from dose–response curves by isobologram analysis. We chose to evaluate anti-proliferative effects using the IC50 for single drug effects that were determined experimentally, using the data from the dose-response experiments as a starting point. Perifosine (from 5 to 20 µM) and cisplatin (IC50: 100 µM) or pemetrexed (IC50: 22 µM) or gemcitabine (IC50: 2 nM) were administered simultaneously for 24 hours to REN cells in colture. At the end of incubation cells were trypsinized and counted. Representative isobolograms in Figure 5 indicate that the combination of perifosine and cisplatin displayed strong synergistic cytotoxicity in REN cells across a broad range of doses. Moreover, perifosine showed additivity in combination with gemcitabine while it seemed to antagonize the effect of pemetrexed. Sinergy with cisplatin is also reported in the MSTO-211H cell line (Figure S1) even if different sensitivity to drugs was observed.

## Discussion

PI3-K/AKT signalling has been demonstrated to have a crucial biologic impact in MMe cell-cycle regulation, anti-apoptosis and chemo-resistance. Activation of AKT, as reported by Altomare et al., was observed in 65% of MMe specimens even if genetic alterations seemed to be infrequent for PI3-K/AKT activation in MMe cells [12].

Moreover, phospho-RTK array analysis showed that in MMe cells the EGFR family, EGFR, ErbB2 or ErbB3, was frequently co-activated with MET, which suggested that MET and EGFR family activation may compensate each other for the persistent downstream signalling activation [28]. In fact, as reported in several studies, even if EGFR and MET were highly expressed in MMe, single RTK inhibitors applied in phase II trials were not sufficient to inhibit MMe cell proliferation, suggesting that inhibition of multiple RTKs may serve to develop a more effective target therapy for patients with MMe in the future [28].

Consideration of PI3K/PDK1/AKT signalling pathway, as a point of convergence of growth factor-related effects on cell proliferation, as a potential new target for MMe therapy, led to the experiments reported here.

Perifosine [octadecyl-(1,1-dimethyl-piperidinio-4-yl)-phosphate] is a synthetic novel alkylphospholipid, a new class of antitumor agents which targets cell membranes, inhibits AKT activation, and induces apoptosis in different carcinoma cells.

The experiments presented in this paper demonstrate that perifosine, caused inhibition of MMe cell growth (IC_50_ by 7.5 µM to 23 µM in 24 hours) and cell cycle arrest at G2-M phase, associated with rapidly decreased AKT activation, as assessed by Ser473 phosphorylation quantification. As preferentially acting on the membrane structure of active proliferating cells, perifosine resulted only slightly toxic on normal mesothelial cells.

The AKT family has three highly homologous isoforms: AKT 1, AKT 2 and AKT 3, encoded by three different genes. All three AKT proteins contain an N-terminal pleckstrin homology(PH) domain, a catalytic kinase domain and a C-terminal regulatory domain.

As the phospho-specific and the anti AKT antibodies we used don't discriminate between the three isoforms, we more deeply investigated their expression in our cells. We firstly describe that MMe cells express AKT1 and AKT3 and that perifosine interferes with both. Considering the tumorigenesis potential of AKT isoforms, further study on function of them in MMe are needed. Inhibition of AKT phosphorylation and cell proliferation were substantially relieved by introduction of a constitutive active myristoylated AKT1 or 3, which bypassed the requirement for PH domain-mediated membrane recruitment, supporting the hypothesis that perifosine does not directly affect activity of PI3-K or phosphoinositide-dependent kinase 1 (PDK1). It has been reported that perifosine enhances the antitumor activity of cetuximab in PTEN-deficient cancer cells and that the combination treatment enhanced the inhibition of phosphorylation of AKT, p44/42MAPK and p38MAPK, but offset the phosphorylation of SAPK/JNK that was activated by perifosine treatment alone [29]. Moreover, it has been described that perifosine showed synergistic effects with erlotinib restoring the efficacy against EGFR inhibitors in PCa [28].

Importantly, when we analysed more deeply the response of MMe cells to EGF and HGF we observed that perifosine, probably acting on cell membrane structure or affecting receptor interactions or recruitment of co-activators, abrogated upstream to signalling molecules both EGFR and MET ligand induced phosphorylation.

Finally we tested the effects of combined therapy with perifosine and cisplatin or pemetrexed or gemcitabine using the isobologram analysis. As evidenced by our results perifosine clearly synergized with cisplatin treatment and exerted an additive effect with gemcitabine.

In summary, we have highlighted AKT, a multifunctional kinase, whose activation is associated with resistance to cell death and tumorigenesis, as an important target of perifosine. We have demonstrated here that perifosine inhibits either AKT1 than AKT3 phosphorylation and activation in cells growing exponentially in serum-containing medium and after growth factor stimulation. This latter mechanisms provide a novel rationale to consider perifosine also as a multi-target inhibitor, whereas interference with the proper membrane localization of target proteins for their phosphorylation and activation (instead of direct inhibition of the phosphotransferase activity of the kinases known to regulate AKT), seems to be a further novel mechanism of the drug's action we disclose here. Although the efficacy of combined treatment between cisplatin and perifosine must be validated by further *in vivo* studies, our results underline perifosine as a multi-target therapy for MMe. More interestingly, perifosine was already shown to give partial response and stable disease in patients in second line therapy (unpublished data kindly provided by Keryx Biopharmaceutical).

## Materials and Methods

### Cell cultures treatments and transfection

The epithelioid MMe derived REN cell line that was used as the principal experimental model in this investigation was kindly provided by Dr. S.M. Albelda (University of Pennsylvania, Philadelphia, PA), MMP were stabilized from pleural effusions of malignant mesothelioma patients [30] and primary HMC-TERT cultures obtained from patients with congestive heart failure and immortalized by expression of a human telomerase subunit [31]. The biphasic derived MSTO-211H cell line was obtained from the Istituto Scientifico Tumori (IST) Cell-bank, Genoa, Italy. Cells were cultured in standard conditions.

Cells grown to 80% confluence in tissue culture dishes were transiently transfected with pcDNA3 Myr-HA-AKT1 or pBABE puroL Myr-HA-AKT3 plasmid encoding for myristilated constitutively active form of AKTs from Addgene by the LipofectAMINE reagent as described by the manufacturer.

### Reagents and antibodies

The monoclonal antibodies specific for phospho tyrosine, phospho-ERK1 (pThr202 and pTyr204) and ERK2 (pThr185 and pTyr187) MAP kinases, and phospho-Akt (pSer473) AKT1 and AKT3, were from Cell Signaling Technology (Beverly, MA). The antibodies specific for α-tubulin, PARP1, ERK1/2, panAKT, MET, EGFR and HA were from Santa Cruz Biotechnology (Santa Cruz, CA). Anti mouse and anti rabbit IgG peroxidase conjugated antibodies, growth factors and chemical reagents were from Sigma–Aldrich (St Louis, MO). ECL was from Amersham Pharmacia Biotech (Uppsala, Sweden). Nitrocellulose membranes and protein assay kits were from Bio-Rad (Hercules, CA). Culture media, sera, antibiotics and LipofectaMINE were from Invitrogen (Carlsbad, CA). Perifosine is marketed and kindly provided by Keryx Biopharmaceutical (New York, NY).

### Cell lysis, immunoprecipitation and immunoblot

Proteins were extracted and immunoprecipitated and SDS-PAGE separated as previously described [32]. Following SDS-PAGE, proteins were transferred to nitrocellulose, reacted with the indicated specific antibodies and then detected with horseradish peroxidase-conjugated secondary antibodies and the chemioluminescent ECL reagent. Densitometric analysis was performed using the GS 250 Molecular Imager (Bio-Rad).

### Cell proliferation as determined by direct counting

MMe cells were grown and treated in complete medium as above indicated, then were trypsinized and stained with Trypan blue. The number of viable cells was counted in a Burker chamber.

### Cell Cycle Analysis

Cell cycle/apoptosis analyses were performed using propidium iodide staining with subsequent FACS analysis. 5×10^5^ cells/well were cultured on tissue culture plates with or without treatment for 24 hours. After incubation, adherent cells were detached with trypsin (0.5% trypsin/0.1% EDTA in PBS). Detached and suspended cells were harvested in complete DMEM medium and centrifuged at 500 g for 10 minutes. Pellets were washed with PBS and fixed with ice cold 75% ethanol overnight at 4°C, treated with 100 µg/ml RNAse A, and subsequently stained with 25 µg/ml propidium iodide. Then they were analyzed by using a flowcytometer FACS (Becton Dickinson, Franklin Lakes, NJ) and Modfit software (Verity Software House, Inc., Topsham, Maine).

### RNA isolation and RT-PCR

Total RNA was extracted from REN, MSTO-211H and A549 cell lines subsequent to the Trizol Reagent (Life Technologies, Inc.) protocol. The quality and quantity of the RNA was determined by measuring the absorbance of the total RNA at 260 and 280 nm. To obtain cDNA was used RevertAid cDNA Synthesis Kit from Fermentas. Primers for Akt isoform expression were described by Okano et al. [33] and were synthesized by MWG. The primers were: 5′-GCTGGACGATAGCTTGGA-3′ (Akt1 sense); 5′-GATGACAGATAGCTGGTG-3′ (Akt1 antisense); 5′-GGCCCCTGATCAGACTCTA-3′ (Akt2 sense); 5′-TCCTCAGTCGTGGAGGAGT-3′ (Akt2 antisense); 5′-GCAAGTGGACGAGAATAAGTCTC-3′ (Akt3 sense); and 5′-ACAATGGTGGGCTCATGACTTCC-3′ (Akt3 antisense). β-Actin primers were 5′-GTGGGGCGCCCCAGGCACCA-3′ (sense) and 5′-CTCCTTAAGTCACGCACGATTTC-3′ (antisense). RT-PCR reactions was performed using 2xPCR Master Mix from Fermentas according standard procedures. The PCR products were electrophoresed on a 1% agarose gel and visualized with ethidium bromide. The Akt primers were designed to generate 383− (Akt1), 276− (Akt2), and 329− (Akt3) bp products, respectively.

### Statistical and Isobologram analysis

Data are expressed as the mean ± SEM. Differences between values obtained in a population of cells treated with different experimental conditions were determined using the unpaired *t*-test. A *P*-value of <0.05 was considered statistically significant. The theoretical basis of the isobologram method has been described in previous studies [34], [35].

Based on the dose–response curves of cisplatin, pemetrexed and gemcitabine, three isobolograms were constructed by plotting the IC50 values of the single drugs on the *y* and of perifosine on the *x* axes respectively, and the theoretical additive dose combination was calculated.

## Supporting Information

Figure S1 **Analysis of perifosine effects on MSTO-211H cells.** A, effect of perifosine on the viability of MSTO-211H cells at 24, 48 and 72 hours treatment at the indicated concentrations. *Points*, means ± SD of three individual measurements. B, Exponentially growing MSTO-211H cells were pre-treated with 20 µM perifosine for 1 hour and then incubated 10 minutes with EGF (5 ng/ml) or HGF (5 ng/ml). At the end of experiment, cell lysates were prepared and analyzed by immunoblotting with indicated antibodies. Results are representative of three different experiments. C, AKT1, 2 and 3 and actin, as control, mRNA expression in MSTO-211H cells was evaluated by RT-PCR as reported in “Material and Methods” section. **D**, Isobologram plot of the interactions between perifosine and cisplatin on MSTO-211H cells.
(TIF)
