# Supplementary figures and images for: Perifosine as a Potential Novel Anti-Cancer Agent Inhibits EGFR/MET-AKT Axis in Malignant Pleural Mesothelioma
Source: PLoS One. 2012 May 10;7(5):e36856. doi: 10.1371/journal.pone.0036856 (PMC3349630; doi:10.1371/journal.pone.0036856)

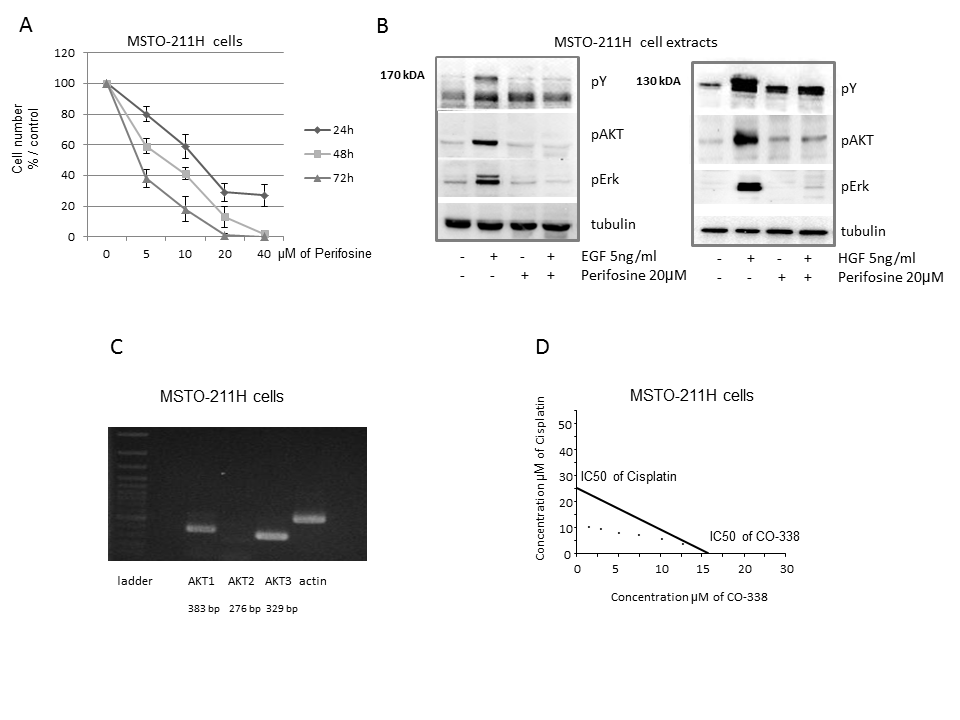

Supplement: Figure S1 — Analysis of perifosine effects on MSTO-211H cells. A, effect of perifosine on the viability of MSTO-211H cells at 24, 48 and 72 hours treatment at the indicated concentrations. Points, means ± SD of three individual measurements. B, Exponentially growing MSTO-211H cells were pre-treated with 20 µM perifosine for 1 hour and then incubated 10 minutes with EGF (5 ng/ml) or HGF (5 ng/ml). At the end of experiment, cell lysates were prepared and analyzed by immunoblotting with indicated antibodies. Results are representative of three different experiments. C, AKT1, 2 and 3 and actin, as control, mRNA expression in MSTO-211H cells was evaluated by RT-PCR as reported in “Material and Methods” section. D, Isobologram plot of the interactions between perifosine and cisplatin on MSTO-211H cells. (TIF) [file pone.0036856.s001.tif]
